# Die neue Weiterbildungsordnung – eine Herausforderung für die Viszeralchirurgie: Ergebnisse einer Umfrage unter DGAV-Mitgliedern und Lösungsstrategien der Jungen Chirurgie

**DOI:** 10.1007/s00104-024-02082-1

**Published:** 2024-04-26

**Authors:** Josefine Schardey, Florentine Hüttl, Anne Jacobsen, Stefanie Brunner, Verena Tripke, Ulrich Wirth, Jens Werner, Jörg C. Kalff, Nils Sommer, Tobias Huber

**Affiliations:** 1 Klinik für Allgemein‑, Viszeral- und Transplantationschirurgie, LMU Klinikum, Ludwig-Maximilians-Universität München, München, Deutschland; 2 Klinik für Allgemein‑, Viszeral- und Transplantationschirurgie, Universitätsmedizin der Johannes Gutenberg-Universität Mainz, Langenbeckstraße 1, 55131 Mainz, Deutschland; 3 Klinik für Allgemein- und Viszeralchirurgie, Friedrich-Alexander-Universität Erlangen-Nürnberg, Erlangen, Deutschland; 4 Klinik für Allgemein‑, Viszeral‑, Tumor- und Transplantationschirurgie, Universitätsklinikum Köln, Köln, Deutschland; 5 Klinik für Chirurgie, Universitätsklinikum Bonn, Bonn, Deutschland; 6 Chirurgische Arbeitsgemeinschaft Junge Chirurgie (CAJC), Deutschen Gesellschaft für Allgemein- und Viszeralchirurgie (DGAV), Berlin, Deutschland

**Keywords:** Viszeralchirurgie, Chirurgische Weiterbildung, Kompetenzbasierte Ausbildung, Umfrage, Training, Visceral surgery, Surgical training, Competency-based education, Survey, Residency

## Abstract

**Hintergrund:**

Die neue kompetenzbasierte Weiterbildungsordnung (nWBO) für chirurgische Weiterbildungen wurde mittlerweile von allen deutschen Landesärztekammern übernommen.

**Methoden:**

Von Mai bis Juni 2023 führte die Arbeitsgemeinschaft Junge Chirurgie (CAJC) eine anonymisierte Onlineumfrage unter den 5896 Mitgliedern der Deutschen Gesellschaft für Allgemein- und Viszeralchirurgie (DGAV) durch.

**Ziel:**

Ziel der Arbeit war es, die Erwartungen an die nWBO zu erfassen und Lösungsstrategien zur Verbesserung der chirurgischen Weiterbildung zu entwickeln.

**Ergebnisse:**

488 Teilnehmende (Rücklaufquote 8,3 %) bedeuten eine repräsentative Studie. Die Befragten setzten sich aus 107 Weiterbildungsassistent:innen (WBA 21,9 %), 69 Fachärzt:innen und 188 Oberärzt:innen (FÄ 14,1 % und OÄ 38,5 %) sowie 107 Chefärzt:innen (21,9 %) zusammen. Die Mehrheit war in Regelversorgern (44 %) tätig, gefolgt von Maximalversorgern (26,8 %) und Universitätsklinika (20,1 %). Nur 22 % halten das geforderte operative Spektrum der nWBO für realistisch. Die Hälfte der Befragten gibt an, dass die volle Weiterbildung in ihrer Klinik gemäß dem neuen Katalog nicht mehr möglich sein wird. 54,6 % halten eine Erreichbarkeit der Richtzahlen in 6 Jahren für unmöglich bzw. geben an, nicht mehr die gleiche Anzahl von WBAs in der gleichen Zeit ausbilden zu können. Einheitlich über alle Versorgungsstufen wurde die Endoskopie (17,1–18,8 %), Fundoplikationen (15,4–17,7 %) und Kopf-Hals-Eingriffe (12,1–17,1 %) als Engstellen genannt. Rotationen wurden laut Angaben bereits zu 64,7 % etabliert. 48 % gaben an, dass in der Abteilung das Teilschrittekonzept etabliert sei. Die Bedeutung eines strukturierten Weiterbildungskonzepts wurde von 85 % der WBA als wichtig erachtet, im Vergleich zu 53,3 % der CÄ. Lag ein strukturiertes Weiterbildungskonzept in der Abteilung vor, so wurde die Erreichbarkeit der Richtzahlen in der univariaten Analyse signifikant positiver eingeschätzt. In der multivariaten Analyse waren das männliche Geschlecht sowie der Status „habilitiert/Professor:in“ unabhängige Faktoren für eine positivere Einschätzung der nWBO. Eine objektive Zertifizierung der Weiterbildung wurde von 51,5 % als wichtig angesehen.

**Schlussfolgerung:**

Die nWBO bereitet Sorgen und die Stimmung ist pessimistisch. Zusätzliche Vorgaben und Krankenhausreformen könnten die Lage verschärfen. Kooperationen und Rotationen sind entscheidend, aber noch nicht ausreichend umgesetzt. Qualitätsorientierte Zertifizierungen könnten die Ausbildungsqualität verbessern.

## Abstract (en)

**Background:**

The new competency-based further training regulations (nWBO) for surgical training have been adopted by all German state medical associations.

**Methods:**

From May to June 2023 the Young Surgeons’ Working Group (CAJC) conducted an anonymous online survey among the 5896 members of the German Society for General and Visceral Surgery (DGAV).

**Objective:**

The survey aimed to assess expectations regarding the nWBO and to develop strategies for enhancing surgical training.

**Results:**

With 488 participants (response rate 8.3%) the study is representative. The respondents consisted of 107 continuing education assistants (WBA 21.9%), 69 specialist physicians, and 188 senior physicians (specialist physicians 14.1% and senior physicians 38.5%), as well as 107 chief physicians (21.9%). The majority worked in regular care providers (44%), followed by maximum care providers (26.8%) and university clinics (20.1%).

Only 22% considered the required operative spectrum of the new medical specialist training regulations (nWBO) to be realistic. Half of the respondents believed that full training in their clinic according to the new catalog will no longer be possible and 54.6% considered achieving the target numbers in 6 years to be impossible or state that they can no longer train the same number of continuing education assistants (WBAs) in the same time frame. Endoscopy (17.1–18.8%), fundoplication (15.4–17.7%) and head and neck procedures (12.1–17.1%) were consistently mentioned as bottlenecks across all levels of care. Rotations for balance were reported to be already established or not necessary in 64.7%. In 48% it was stated that the department had established the partial steps concept.

The importance of a structured training concept was considered important by 85% of WBAs, compared to 53.3% of chief physicians (CÄ). If a structured training concept was present in the department, the achievability of the target numbers was significantly assessed more positively in the univariate analysis. In the multivariate analysis, male gender and the status of “habilitated/professor” were independent factors for a more positive assessment of the nWBO. Objective certification of training was considered important by 51.5%.

**Conclusion:**

Concerns surround the nWBO and the sentiment is pessimistic. Additional requirements and hospital reforms could exacerbate the situation. Collaboration and rotations are crucial but still insufficiently implemented. Quality-oriented certification could enhance the quality of training.

## Hintergrund

Die auf dem Deutschen Ärztetag 2018 beschlossene neue Muster-Weiterbildungsordnung (MWBO) ist 2021 in Kraft getreten und wurde mittlerweile in allen Bundesländern in die jeweilige Landesärztekammer übernommen und als jeweilige Weiterbildungsordnung (nWBO) mit nur wenigen Veränderungen übernommen [1]. Die nWBO soll eine vermehrt kompetenzbasierte Weiterbildung ermöglichen. Die meisten Übergangsregelungen laufen Ende 2023 aus [29].

Patientensicherheit, Versorgungsqualität und wirtschaftliche Anforderungen sind heutzutage für Leistungserbringer, Interessengruppen und die Öffentlichkeit von großer Relevanz [26]. Im Spannungsfeld zwischen medizinischer Versorgung und Weiterbildung besteht das übergeordnete Ziel darin, den angehenden Chirurg:innen das erforderliche Wissen und die notwendigen Fähigkeiten für ihre zukünftige Tätigkeit zu vermitteln [26]. Die Chancen, manuell-technische Fähigkeiten zu erwerben, scheinen seit der Einführung von Arbeitszeitvorschriften gesunken zu sein. Angemessene Bildungsmöglichkeiten müssen demnach effektiver genutzt werden, um sicherzustellen, dass diese Fertigkeiten auf einem hohen Niveau erhalten bleiben [9]. Deshalb ist ein Wechsel von der reinen Anzahl der durchgeführten Eingriffe hin zu einer kompetenzbasierten Ausbildung in der chirurgischen Weiterbildung durchaus sinnvoll. Bislang wurde diese jedoch nicht angemessen kontrolliert. Durch genauere Definierung der zu vermittelnden Inhalte und die Einführung kontinuierlich geführter E‑Logbücher ergeben sich in Zukunft diesbezüglich bessere Möglichkeiten [7].

In dieser deutschlandweiten Umfrage unter den Mitgliedern der deutschen Gesellschaft für Allgemein- und Viszeralchirurgie (DGAV) sollte eine Einschätzung der nWBO ermittelt und basierend auf den Umfrageergebnissen potenzielle Lösungsstrategien für die Optimierung der chirurgischen Weiterbildung skizziert werden.

## Material und Methoden

### Rekrutierung

Nach Einführung der nWBO wurde unter den Mitgliedern der DGAV eine anonymisierte Onlineumfrage durchgeführt. Sensible personenbezogene Daten wurden nicht erfasst und ethische Richtlinien (Deklaration von Helsinki) wurden eingehalten. Die Onlineumfrage wurde mit dem Tool Lime-Survey ©Community Edition Version 5.2.6 (LimeSurvey GmbH, Hamburg, Deutschland) erstellt. Die Installation wurde auf einem Server der Johannes Gutenberg-Universität Mainz gehostet, um ausreichenden Datenschutz zu gewährleisten. Enthalten waren Fragen zur Einschätzung der nWBO sowie zur zukünftigen Gestaltung der chirurgischen Weiterbildung. Der Fragebogen ist online einsehbar unter https://survey.zdv.uni-mainz.de/index.php/728827?lang=de. Es erfolgten zweimalige E‑Mail-Aussendungen über den DGAV-Verteiler am 11.04.2023 und am 10.05.2023 an die 5896 Mitglieder der DGAV. Die Fallzahlplanung ergab bei einem gewünschten Konfidenzniveau von 95 % für die gewünschte Fehlermarge (0,05) bei in der Planungsphase angenommenen 5900 Mitgliedern der DGAV eine benötigte Stichprobengröße von *n* = 385.

### Statistik

**Abb. 1:**
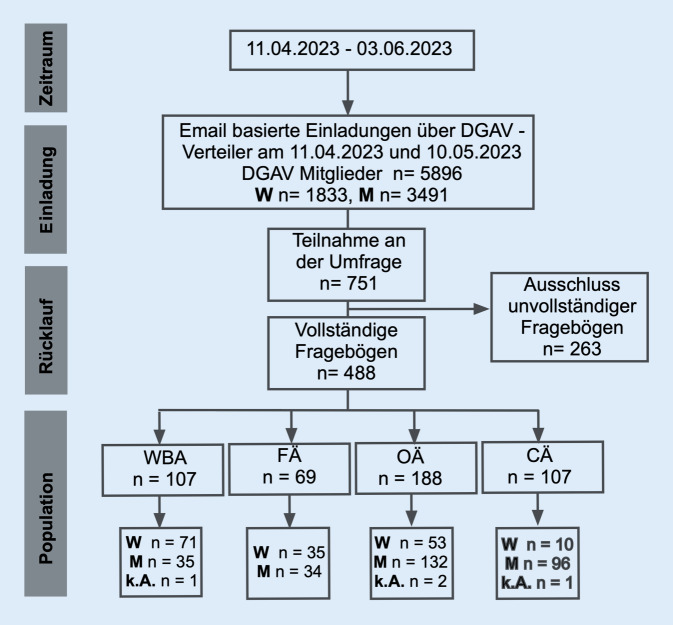
Consort-Diagramm: Herkunft der Teilnehmenden der deutschlandweiten Umfrage unter Mitgliedern der DGAV von April bis Mai 2023. *CÄ* Chefärztinnen und Chefärzte, *OÄ* Oberärztinnen und Oberärzte, *FÄ* Fachärztinnen und Fachärzte, *WBA* Ärztinnen und Ärzte in Weiterbildung

Unvollständige Fragebögen wurden ausgeschlossen (Abb. 1). Die Daten wurden mit SPSS Version 25 (IBM, USA) und Graph-Pad Prism 9 (GraphPad Software Inc., USA) analysiert. Deskriptive Statistiken wurden je nach Stichprobengröße unter Verwendung des χ^2^- oder Fishers-exact-Test durchgeführt. In der multivariaten Analyse kam eine binär logistische Regressionsanalyse zum Einsatz. *p*-Werte < 0,05 wurden als signifikant betrachtet.

## Ergebnisse

### Rücklaufquote

**Abb. 2:**
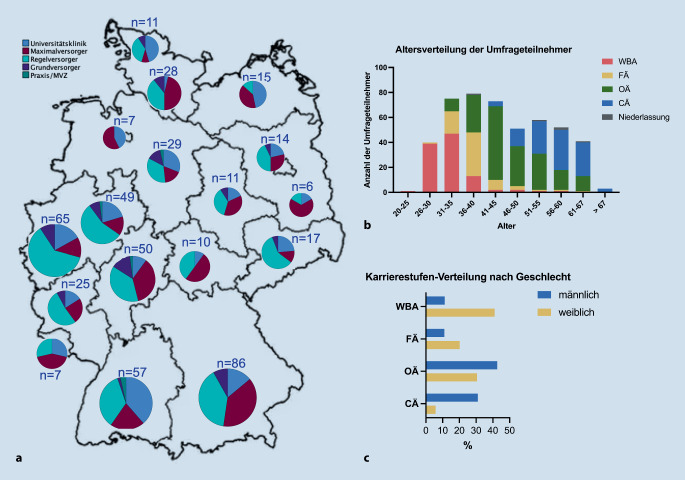
Landesärztekammerzugehörigkeit und Versorgungsstufe (**a**) sowie Altersverteilung (**b**) und Verteilung zwischen männlichen und weiblichen Teilnehmer:innen (**c**). *CÄ* Chefärztinnen und Chefärzte, *OÄ* Oberärztinnen und Oberärzte, *FÄ* Fachärztinnen und Fachärzte, *WBA* Ärztinnen und Ärzte in Weiterbildung

Insgesamt wurden 488 vollständige Fragebögen analysiert (Abb. 1). Am 10.05.2023 betrug die Anzahl der Mitglieder nach Auskunft der DGAV Geschäftsstelle *n* = 5896. Damit beläuft sich die Rücklaufquote auf 8,3 %. Die Teilnehmenden gehörten überwiegend der Bayerischen Landesärztekammer (17,6 %), den Landesärztekammern Nordrhein (13,3 %) und Westfalen-Lippe (10,0 %) sowie den Landesärztekammern Baden-Württemberg (11,7 %) und Hessen (10,2 %) an (Abb. 2a).

### Charakteristika der Teilnehmenden

Die größte Gruppe befand sich in der Altersklasse von 36 bis 40 Jahren (16,0 %), gefolgt von den 31- bis 35-Jährigen (15,4 %) und den 41- bis 45-Jährigen (15,0 %, Abb. 2b). Die Mehrheit gab an, in einem Regelversorger (RV) zu arbeiten (43,9 %), gefolgt von einem Maximalversorger (MV) (26,8 %), einer Universitätsklinik (UK 20,0 %) und einem Grundversorger (GV, 7,6 %). Ein kleiner Anteil war in einem Medizinischen Versorgungszentrum (MVZ) oder in einer Praxis (niedergelassene Chirurgie [NC]) tätig (1,4 %). Knapp 34 % befanden sich in Weiterbildung, 38,5 % waren oberärztlich und 21,9 % chefärztlich tätig.

Der Anteil männlicher Teilnehmer war in RV mit knapp 70 % am höchsten, diese waren auch in anderen Versorgungsstufen mit knapp 60 % vermehrt vertreten. Eine detaillierte Aufschlüsselung finden sich in Tab. 1. Der Anteil von Frauen nimmt mit höheren beruflichen Positionen ab (Abb. 2c).

**Tab. 1:** Charakteristika der Umfrageteilnehmer und Arbeitsstellen

Institution	UK *N* (%)		MV *N* (%)		RV *N* (%)		GV *N* (%)		NC *N* (%)		k.A
*Gesamt N* *=* *488*	98	(20,1)	131	(26,8)	214	(43,9)	37	(7,6)	7	(1,4)	1
*Alter*											
< 40 Jahre	62	(63,3)	62	(47,3)	59	(27,6)	12	(32,4)	1	(14,3)	–
> 40 Jahre	36	(36,7)	67	(51,1)	154	(72,0)	25	(67,6)	6	(85,7)	1
*Geschlecht*											
Männlich	58	(59,2)	76	(58,0)	149	(69,6)	23	(62,2)	4	(57,1)	–
Weiblich	38	(38,8)	54	(41,2)	63	(29,4)	14	(37,8)	3	(42,9)	1
k. A.	1	(1,0)	1	(0,8)	2	(1)	–	–	–	–	–
*Arbeitsmodell*											
k. A.	1	(1,0)	2	(1,5)	2	(0,9)	–	–	–	–	–
Vollzeit	92	(93,9)	114	(87,0)	179	(83,6)	32	(86,5)	4	(57,1)	–
Teilzeit	4	(4,1)	10	(7,6)	28	(13,1)	5	(13,5)	3	(42,9)	1
Elternzeit	1	(1,0)	4	(3,1)	4	(1,9)	–	–	–	–	–
Sonstiges	–	–	1	(0,8)	1	(0,5)	–	–	–	–	–
*Betreuung Angehöriger*	40	(48)	55	(42)	101	(47,2)	19	(51,4)	3	(42,9)	1
*Abschluss/akademischer Grad*											
Approbation	11	(11,2)	39	(29,8)	65	(30,3)	9	(24,3)	1	(14,3)	1
Promotion	49	(50,0)	62	(47,3)	120	(56,1)	22	(59,5)	4	(57,1)	–
Habilitation	17	(17,3)	12	(9,1)	11	(5,1)	–	–	2	(28,6)	–
Professur	21	(21,4)	11	(8,3)	15	(7,0)	2	(5,4)	–	–	–
k. A.	–	(11,2)	2	(1,5)	1	(0,4)	2	(5,4)	–	–	–
*Berufliche Position*											
CÄ	10	(10,2)	21	(16,0)	60	(28,0)	15	(40,5)	1	(14,3)	–
OÄ	39	(39,8)	41	(31,3)	94	(43,9)	12	(32,4)	1	(14,3)	1
FÄ	21	(21,4)	29	(22,1)	13	(6,1)	5	(13,5)	1	(14,3)	–
WBA	27	(27,6)	37	(28,2)	38	(17,8)	5	(13,5)	–	–	–
Niederlassung	–	–	–	–	1	(0,5)	–	–	4	(57,1)	–
*Strukturierte Weiterbildung in der Abteilung?*											
Ja	44	(44,9)	65	(49,6)	125	(58,4)	23	(62,2)	2	(28,6)	–
Nein	44	(44,9)	56	(42,7)	66	(30,8)	9	(24,3)	3	(42,9)	1
Geplant	9	(9,2)	7	(5,3)	12	(5,6)	5	(13,5)	1	(14,3)	–
Weiß ich nicht	1	(1,0)	3	(2,3)	11	(5,1)	23	(62,2)	1	(14,3)	–
*Mögliche Weiterbildungszeit Viszeralchirurgie:*											
Volle Weiterbildung	97	(99,0)	122	(93,1)	138	(64,5)	15	(40,5)	–	–	1
36–48 Monate	–	–	4	(3,1)	33	(15,4)	8	(21,6)	–	–	–
24–36 Monate	–	–	1	(0,8)	16	(7,5)	2	(5,4)	–	–	1
12–24 Monate	–	–	2	(1,5)	14	(6,5)	10	(27,0)	1	(14,3)	–
12 Monate	–	–	–	–	4	(1,9)	–	–	2	(28,6)	–
Weiß ich nicht	–	–	122	(93,1)	6	(2,8)	2	(5,4)	4	(57,1)	–
k. A.	–	–	2	–	3	(1,4)	–	–	–	–	–

*UK* Universitätsklinik, *MV* Maximalversorger, *RV* Regelversorger, *GV* Grundversorger, *NC* Niedergelassene Chirurgie, *k. A.* keine Angabe

Der Anteil der Teilzeitbeschäftigung war höher in Kliniken der GV (13,5 %, 0 % in Elternzeit) und RV (13,1 %, 1,9 % in Elternzeit, Tab. 1). An UK betrug der Anteil der Vollzeitarbeitenden knapp 94 %, und nur 4,1 % arbeiteten in Teilzeit (1 % in Elternzeit). Bei den MV zeigte sich, dass bereits ein größerer Anteil (7,6 %) in Teilzeit arbeitete (1 % in Elternzeit). Unter den Teilnehmenden stellten Oberärzte (OÄ) mit 188 Teilnehmenden die größte Gruppe dar (38,5 %), gefolgt von insgesamt 107 Chefärzten (CÄ, 34 %), von denen die Mehrheit in einer RV tätig war. Die Gruppe von 69 Fachärzten (FÄ) arbeitete überwiegend an UK oder in MV, während die Weiterbildungsassistenten (WBA) mit 107 Teilnehmenden am häufigsten aus MV stammten (21,7 %). An einer UK war die volle Weiterbildung möglich, 93,1 % arbeiten in MV, wo dies ebenfalls möglich ist.

### Engstellen und Kooperationen

#### Engstellen

**Abb. 3:**
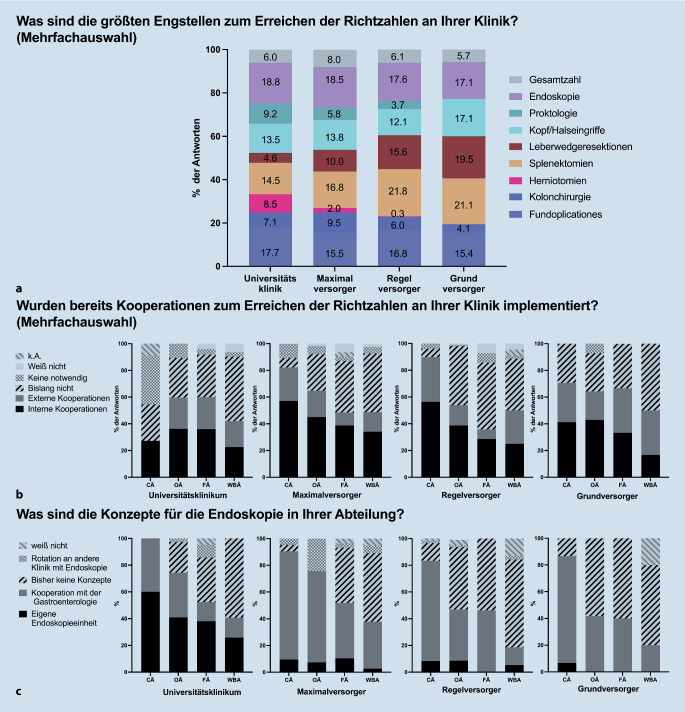
Darstellung der Mehrfachantworten zu potenziellen Engstellen zur Umsetzung der neuen WBO nach Eingriffen und Standort (**a**), mögliche Lösungsstrategien im Hinblick auf Kooperationen (**b**) und Lösungsstrategien für die Endoskopie (**c**). *CA* Chefärztinnen und Chefärzte, *OÄ* Oberärztinnen und Oberärzte, *FÄ* Fachärztinnen und Fachärzte, *WBA* Ärztinnen und Ärzte in Weiterbildung

Die Endoskopie wurde mit einem Anteil von 17,1–18,8 % in allen Versorgungsstufen als größte Herausforderung angesehen, gefolgt von Fundoplikationen mit 15,4–17,7 %. Aber auch die Kopf-Hals-Eingriffe wurden in 12,1–17,1 % der Antworten genannt. Die Proktologie wurde in 9,1 % der Antworten an UK als problematisch angesehen, während dies bei den GV nicht der Fall war. Ein ähnliches Muster zeigte sich bei Herniotomien, die mit 8,5 % der Antworten an UK genannt wurden. Die Kolonchirurgie wurde immerhin von 9,5 % an MV tätigen Ärzt:innen genannt. Die Nennung der Leberwedgeresektionen stieg von 4,6 % an UK bis auf 19,5 % bei den GV an, ebenso wie die Splenektomien von 14,5 % auf über 21 % bei den GV und RV (Abb. 3a).

#### Kooperationen

Insgesamt gaben 40,5 % an, dass bereits eine interne und 21,8 %, dass eine externe Kooperation etabliert sei. 2,4 % gaben an, dass keine notwendig sei. In 32,5 % wurde angeben, dass bislang keine Kooperationen etabliert seien (Abb. 3b).

#### Endoskopie

Die Konzepte für die Endoskopie unterschieden sich je nach Position und Versorgungsstufe. In UK gaben 60 % der CÄ (*n* = 10) an, über eine eigene Endoskopieeinheit zu verfügen, während 40 % eine Kooperation mit der Gastroenterologie angaben. Bei den OÄ und FÄ, die an UK tätig waren, gaben 41 und 38 % an, über eine eigene Endoskopieeinheit zu verfügen (Abb. 3c).

Im Kontrast dazu gaben CÄ in MV, GV und RV in mehr als 80 % der Fälle an, dass sie eine Kooperation mit der Gastroenterologie als Konzept zu haben. Konträr dazu gaben bei den WBA aller Versorgungsstufen fast 60 % an, dass bisher keine Konzepte vorhanden seien.

### Einschätzung der Richtzahlen

#### Erreichbarkeit der Richtzahlen

**Abb. 4:**
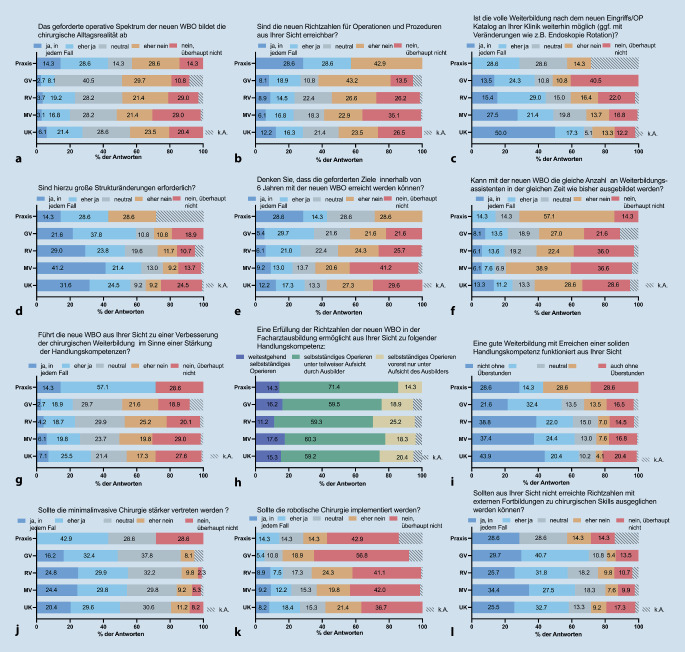
Einschätzung der Folgen der neuen WBO hinsichtlich des Spektrums (**a**), Richtzahlen (**b**), notwendigen Veräderungen (**c**), Strukturänderungen (**d**), Ziele (**e**), Zeithorizont (**f**), Handlungskompetenz (**g**–**i**), Minimalinvasive (**j**) und Robotische Chirurgie (**k**) und Simulation (**l**), aufgetragen nach Versorgungsstufe. *UK* Universitätsklinik, *MV* Maximalversorger, *RV* Regelversorger, *GV* Grundversorger, *NC* Niedergelassene Chirurgie, *k.A.* keine Angabe

Laut Umfrageteilnehmer:innen spiegelt die nWBO nur etwa 22 % des geforderten operativen Spektrums wider (Abb. 4a), und 54 % der Befragten halten die geforderten Richtzahlen für nicht/eher nicht erreichbar (Abb. 4b). Für etwa die Hälfte der Befragten wird die volle Weiterbildung gemäß dem neuen Eingriffs‑/Operationskatalog an ihrer Klinik nicht mehr möglich sein, aber etwa ein Drittel schätzt dies als möglich ein (Abb. 4c). Diese Einschätzung variierte je nach Versorgungsstufe signifikant (*p* = 0,002) und war auch in Abhängigkeit von der beruflichen Position signifikant unterschiedlich (*p* < 0,001), wobei CÄ und OÄ positiver gestimmt sind als WBA.

Etwa 54,4 % der Befragten stimmten zu, dass große Strukturänderungen erforderlich sind, während ein Viertel der Befragten dies ablehnt (Abb. 4d). Dabei glaubten CÄ seltener (31 %) an die Notwendigkeit großer Strukturänderungen als WBA (73 %).

In Bezug auf das Erreichen der Richtzahlen innerhalb von 6 Jahren sind 54 % skeptisch, während 27 % dies für möglich halten (Abb. 4e). Ebenfalls erwarten 62,3 % der Befragten, nicht mehr die gleiche Anzahl von WBA in der gleichen Zeit ausbilden zu können, obwohl kumulativ 20 % immer noch daran glauben (Abb. 4f). Diese Wahrnehmung variierte je nach beruflicher Position signifikant (*p* < 0,001), wobei CÄ, OÄ und FÄ optimistischer sind als WBA.

#### Erreichen einer soliden Handlungskompetenz

Eine Stärkung der Handlungskompetenz durch Umsetzung der nWBO erwarteten kumulativ 26,2 % der Befragten (Abb. 4g), wohingegen sich 45,7 % negativ in diesem Zusammenhang äußerten. Jedoch gaben fast 60 % mit Frage nach dem erwarteten Ziel der Handlungskompetenz an, dass „selbstständiges Operieren unter teilweiser Aufsicht durch den Ausbilder“ erreicht werden können (Abb. 4h).

Überraschende Einigkeit herrschte in Bezug auf Überstunden: Fast 71 % gaben an, dass aus ihrer Sicht eine gute Weiterbildung mit Erreichen einer soliden Handlungskompetenz ohne Überstunden „nicht/eher nicht“ funktioniere, 21 % hielten dagegen (Abb. 4i).

#### Implementierung minimal-invasiver, robotischer Verfahren und Simulationstraining

Die Implementierung der MIC befürworteten 53,3 % der Befragten, während etwa ein Drittel neutral eingestellt war und 15 % dies ablehnten (Abb. 4j). Eine Implementierung der robotischen Chirurgie lehnten hingegen 64,8 % ab, nur knapp 20 % bewerteten dies positiv (Abb. 4k). Bei der Berücksichtigung der beruflichen Position wurde die Implementierung der Robotik von den WBA häufiger positiv (29 %) bewertet als von CÄ (14 %), OÄ (18,1 %) und FÄ (15,9 %), (*p* = 0,005). Insgesamt bewerteten 59,4 % den Ausgleich nicht erreichter Richtzahlen durch den Einsatz externer Fortbildungen, wie Simulationstraining, positiv, während kumulativ 20,1 % dies negativ beurteilten (Abb. 4l).

### Einschätzung verschiedener Lehr‑, Arbeits- und Lösungsstrategien

#### Bedeutung Lehr- und Lernkonzepte

**Abb. 5:**
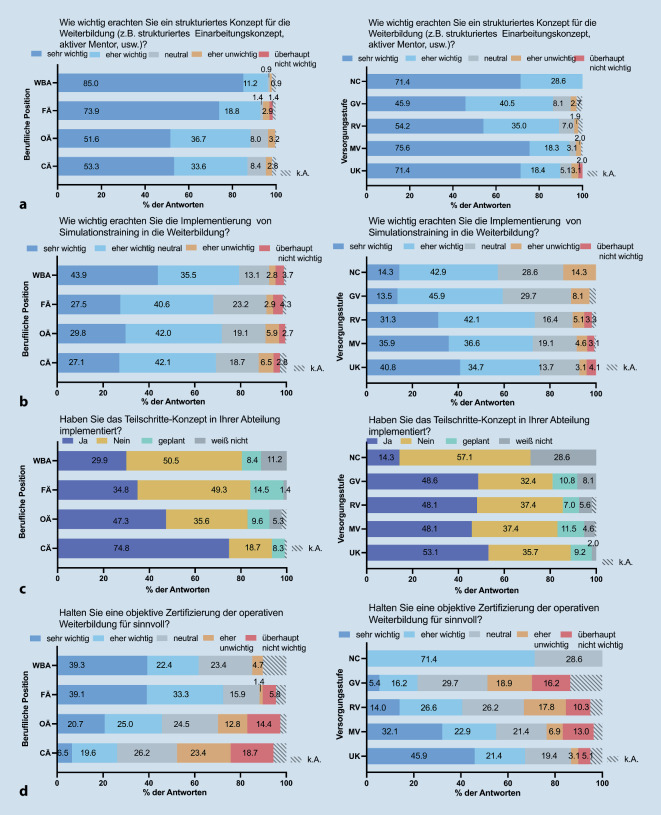
Umfrageergebnisse zu verschiedenen Weiterbildungskonzepten (**a**, **b**), Implementierung des Teilschrittekonzepts (**c**) sowie einer möglichen Weiterbildungszertifizierung (**d**) aufgetrennt nach beruflicher Position (*links*) und Klinikversorgungsstufe (*rechts*). *CÄ* Chefärztinnen und Chefärzte, *OÄ* Oberärztinnen und Oberärzte, *FÄ* Fachärztinnen und Fachärzte, *WBA* Ärztinnen und Ärzte in Weiterbildung, *UK* Universitätsklinik, *MV* Maximalversorger, *RV* Regelversorger, *GV* Grundversorger, *NC* Niedergelassene Chirurgie, *k.A.* keine Angabe

Ein strukturiertes Weiterbildungskonzept war für 85 % der WBA wichtig, im Vergleich zu 53,3 % der CÄ (*p* = 0,107, Abb. 5a). 73,2 % erachteten die „Implementierung von Simulationstraining“ (Abb. 5b) als „wichtig/sehr wichtig“. Die Mehrheit von 91,5 % betonte die Bedeutung eines „fachlich-theoretischen Selbststudiums“ und sowie interner Fortbildungen (79,0 %, Abb. nicht gezeigt).

In 48,1–53,1 % der Kliniken ist ein Teilschrittekonzept implementiert (Abb. 5b). Die Einführung eines Teilschrittekonzepts wurde von 74,8 % der CÄ angegeben, während es bei den WBA nur in 29,9 % der Fälle bestätigt wurde.

#### Zertifizierung der Weiterbildung

Eine objektive Zertifizierung der Weiterbildung wurde von 51,5 % als „wichtig/sehr wichtig“ angesehen, während 25 % neutral und 23,4 % dies für nicht sinnvoll hielten. Es gab signifikante Unterschiede in den Antworten je nach Karrierestufe, wobei OÄ, FÄ und WBA dies überwiegend positiv bewerteten im Vergleich zu CÄ (*p* < 0,001). Auch nach Institution gab es signifikante Unterschiede, dies wurde an UK als wichtiger angesehen als in GV (*p* < 0,001, Abb. 5c).

### Auswertung der offenen Antworten

Gefragt nach den Vorteilen der nWBO äußerten sich nur 58,5 % hierzu positiv (Tab. 2): Vorrangig wurde hierbei eine bessere bzw. breitere Ausbildung angeführt, 8,2 % sahen die Stärkung der Endoskopie positiv. Andere genannte positive Punkte waren eine breitere Ausbildung sowie mehr Transparenz und eine Stärkung der minimal-invasiven Chirurgie (MIC) und eine Annäherung an das Spektrum von Häusern der GV und RV.

**Tab. 2:** *N* = 147 der befragten Personen haben im Freitext die Vorteile der neuen WBO aufgeführt. Diese wurden zusammengefasst und entsprechend ausgewertet

Vorteile der neuen WBO	*N* = 147 Antworten	Prozent der Antworten (in %)
Keine Vorteile/nur Nachteile	61	41,5
Breitere Ausbildung	51	34,7
Stärkung der Endoskopie	12	8,2
Spezifischere Ausbildung	9	6,1
Mehr Transparenz	9	6,1
Stärkung MIC	3	2,0
Anpassung des Spektrum an Grund/Regelversorger	2	1,4

In den offenen Fragen nach möglichen Nachteilen der nWBO (Tab. 3) wurde in 28,2 % der Fälle (d.F.). die Zusammensetzung der geforderten Eingriffe kritisiert sowie eine damit einhergehende Verlängerung der Weiterbildungszeit (20,4 % d.F.). 11 % kritisierten eine Notwendigkeit für Rotationen. 18,2 % führten eine Benachteiligung der GV und RV gegenüber MV und UK an. Die Endoskopie wurde in 16,6 % der Fälle als nicht sinnvoll angegeben.

**Tab. 3:** *N* = 181 Personen haben im Freitext die Nachteile der neuen WBO aufgeführt. Diese wurden zusammengefasst und aufgrund der häufigen Mehrfachnennungen entsprechend Mehrfachantworten ausgewertet

Nachteile der neuen WBO	*N* = 271	Prozent der Antworten (in %)	Prozent der Fälle (in %)
Zusammensetzung der geforderten Eingriffe insgesamt unrealistisch	51	18,8	28,2
Verlängerung der Weiterbildungszeit/Weniger Assistenten in der gleichen Zeit	37	13,7	20,4
Sonstiges	34	12,5	18,8
Benachteiligung der Grund- und Regelversorger gegenüber MV/UK	34	12,5	18,8
Endoskopie nicht sinnvoll	30	11,1	16,6
Notwendigkeit für Rotationen	20	7,4	11,0
Falsche Dokumentation/Bescheinigungen	11	4,1	6,1
Splenektomien unrealistisch	11	4,1	6,1
Abschreckende Wirkung auf Assistenzärzte	10	3,7	5,5
Koloneingriffe unrealistisch	9	3,3	5,0
Erhöhte Bürokratie/Dokumentationsaufwand	8	3,0	4,4
Zahl Fundoplikationen unrealistisch	8	3,0	4,4
Zahl Kopf‑/Halseingriffe unrealistisch	8	3,0	4,4

### Faktoren für eine positive bzw. negative Bewertung der Richtzahlen

Es konnten mehrere Faktoren bestimmt werden, welche die Beurteilung der Erreichbarkeit der geforderten Richtzahlen beeinflussen könnten (Tab. 4). Weibliche Teilnehmerinnen schätzten die Erreichbarkeit pessimistischer ein, mit 82,6 % („nein/eher nein“) im Vergleich zu 59,2 % der männlichen Teilnehmer (*p* < 0,001). Teilnehmer:innen > 40 Jahre schätzten die Erreichbarkeit positiver ein als Teilnehmer:innen < 40 Jahre (*p* < 0,001). Auch die berufliche Position hatte signifikante Auswirkungen, wobei CÄ und OÄ dies positiver bewerteten. In Abteilungen mit strukturierter Weiterbildung wurde die Erreichbarkeit ebenfalls positiver eingeschätzt (*p* = 0,001). Betreuung von Angehörigen hatte keinen Einfluss auf die Antworten (*p* = 0,092), ebenso wenig wie Vollzeit- und Teilzeitbeschäftigung (*p* = 0,575).

**Tab. 4:** Univariate Analyse prädiktiver Faktoren der Umfrageteilnehmer im Hinblick auf eine positive oder negative der Einschätzung der Richtzahlen

Richtzahlen erreichbar	Nein/eher nein	Ja/eher ja	*p*-Wert
	Anzahl d. Antworten (%)	Anzahl d. Antworten (%)	*χ*^2^
*Alter*	–	–	< 0,001*
< 40 Jahre	133 (84,2 %)	25 (15,8 %)	–
> 40 Jahre	130 (58,0 %)	94 (42,0 %)	–
*Geschlecht*	–	–	< 0,001
Weiblich	119 (82,6 %)	25 (17,4 %)	–
Männlich	141 (59,2 %)	97 (40,8 %)	–
*Abschluss*	–	–	< 0,001
Approbation	84 (79,2 %)	22 (20,8 %)	–
Promotion	148 (72,2 %)	57 (27,8 %)	–
Habilitation	11 (35,5 %)	20 (64,5 %)	–
Professur	14 (42,4 %)	19 (57,6 %)	–
*Position*	–	–	< 0,001
CÄ	34 (43,0 %)	45 (57,0 %)	–
OÄ	97 (67,8 %)	46 (32,2 %)	–
FÄ	50 (82,0 %)	11 (18,0 %)	–
WBA	74 (83,1 %)	15 (16,9 %)	–
*Institution*	–	–	0,705
Universitätskliniken	49 (63,6 %)	28 (36,4 %)	–
Maximalversorger	76 (71,7 %)	30 (28,3 %)	–
Regelversorger	113 (69,3 %)	50 (30,7 %)	–
Grundversorger	21 (67,7 %)	10 (32,3 %)	–
*Strukturierte Weiterbildung*			0,001
Ja	123 (59,7 %)	83 (40,3 %)	–
Nein	115 (79,9 %)	29 (20,1 %)	–
Geplant	17 (73,9 %)	6 (26,1 %)	–
Weiß nicht	8 (66,7 %)	4 (33,3 %)	–
*Betreuen Sie derzeit Kinder oder andere Angehörige?*			0,092^a^
Ja	107 (64,1 %)	60 (35,9 %)	–
Nein	147 (72,4 %)	56 (27,6 %)	–
*Arbeiten Sie in Voll- oder Teilzeit*			0,575
Vollzeit	105 (31,7 %)	226 (68,3 %)	–
Teilzeit	12 (29,3 %)	29 (70,7 %)	–
Elternzeit	1 (16,7 %)	5 (83,3 %)	–

^a^Fishers-exact-Test

In der multivariaten Analyse waren das männliche Geschlecht sowie der Status „habilitiert/Professor:in“ unabhängige Faktoren für eine positivere Einschätzung der nWBO (Tab. 5).

**Tab. 5:** Multivariate binär logistische Regressionsanalyse für die positive Einschätzung der Richtzahlen

Variable	Regressionskoeffizient	Standardfehler	Wald	*Freiheitsgrad*	Odds Ratio	95 %-Konfidenzintervall	Binär log. Regression *p*-Wert
*Alter: >* *40 Jahre*	0,720	0,424	2,891	1	2,055	0,896–4,714	0,089
*Position*	–	–	3,255	4	–	–	0,516
CÄ	0,444	0,348	1,626	1	0,642	0,324–1,269	0,202
OÄ	0,801	0,603	1,767	1	0,449	0,138–1,463	0,184
FÄ	0,410	0,631	0,423	1	0,663	0,193–2,286	0,516
WBA	0,532	1,134	0,220	1	1,702	0,184–15.720	0,639
*Habilitiert/Prof*	0,962	0,347	7,673	1	2,617	1,325–5,169	**0,006**
*Männliches Geschlecht*	0,630	0,313	4,046	1	1,877	1,016–3,467	**0,044**
*Strukturierte WB in der Abteilung*	0,139	0,311	0,201	1	1,150	0,625–2,116	0,654

fett: statistisch signifikant

## Diskussion

Die aktuelle Erhebung mit 488 vollständig ausgefüllten Fragebögen übertrifft die erforderliche Größe von 385 Teilnehmenden und gilt daher als repräsentativ für die Mitglieder der DGAV. Im Vorjahr führten wir eine Umfrage unter bayerischen Chirurg:innen und WBA im Fach Allgemein- und Viszeralchirurgie durch, um die Auswirkungen der Einführung der nWBO zu untersuchen [25]. Dabei wurden potenzielle Unterschiede zwischen „jungen Chirurg:innen“ und „Weiterbildenden“ analysiert, und es wurde eine pessimistische Haltung bezüglich der Umsetzbarkeit der nWBO festgestellt [25]. Die Ergebnisse der aktuellen Umfrage bestätigen, dass diese Erkenntnisse auf andere Landesärztekammern übertragbar sind. Nur etwa 22 % der Befragten sehen die geforderten operativen Anforderungen der neuen Weiterbildungsordnung im chirurgischen Alltag abgebildet. Es gibt weiterhin Bedenken auf allen Versorgungsebenen, insbesondere in Bezug auf Eingriffe wie Fundoplikationen, Splenektomien und Kopf-Hals-Eingriffe. Besorgniserregend ist, dass 35–42 % der Teilnehmenden aller Versorgungsstufen angeben, dass bisher keine Kooperationen etabliert wurden. Zusätzlich bestehen Engpässe bei Ausbildungsplätzen in der Endoskopie, obwohl dies ein wichtiger und vor allem auch geforderter Bestandteil der Viszeralchirurgie ist [12]. Die Konzepte für die Endoskopie unterscheiden sich je nach Position und Versorgungsstufe, wodurch der Bedarf klarer Richtlinien für den Erwerb der Kompetenzen ersichtlich wird. Die aktive Beteiligung zahlreicher CÄ, von denen viele die Weiterbildungsbefugnis in ihren Abteilungen innehaben, unterstreicht die Relevanz dieses Themas in der DGAV.

### Limitationen

Die Beantwortung der Umfrage war aufwendig, da Teilnehmer:innen zunächst die alten und neuen Richtlinien studieren mussten, bevor sie ihre Erwartungen äußern konnten. Dies könnte die Teilnahmebereitschaft beeinflusst haben, was sich in den lediglich 65 % vollständig beantworteter Fragebögen widerspiegelt. Jedoch wurde trotz niedriger Teilnahmequote von 8,3 % für eine webbasierte Umfrage die angestrebte Repräsentativität erreicht. Mehrfachbeantwortungen waren theoretisch möglich, aber unwahrscheinlich, da keine Belohnung ausgeschrieben war. Fragen zur kognitiven und Methodenkompetenz wurden nicht gestellt, da in der nWBO keine Nachweise erforderlich sind. Die Gefahr von Antwortverzerrungen wurde als gering eingeschätzt, da die meisten Fragen auf eine Bewertung der Auswirkungen in skalierter Form abzielten. Weiterhin bestand eine geringe Beteiligung von WBA, was auf einen möglichen Selektionsbias hinweisen könnte, da diese Gruppe möglicherweise noch nicht Mitglied der Fachgesellschaft ist. Obwohl der Anteil der WBA in der DGAV kontinuierlich auf ein Drittel gestiegen ist (*n* = 1987, Stand 5/2023), nahmen insgesamt nur 5,4 % teil. Daher kann nur unter Akzeptanz einer leicht erhöhten Fehlerspanne von 0,1 von einer repräsentativen Teilnahme auch innerhalb der Subgruppen ausgegangen werden.

### Weiterbildungszeit

Die CAJC bietet auf der DGAV Website eine Ideensammlung „Weiterbildung To Go“ an [20]. Obwohl sie sich auf die alte WBO bezieht, ist die Grundproblematik weiter gültig, ja sogar durch die nWBO verschärft worden. Die formale Weiterbildungszeit beträgt 6 Jahre, jedoch ist die effektive Zeit für das Operationstraining erheblich kürzer. Es bleiben im Durchschnitt 250 Arbeitstage, abzüglich 30 Urlaubstage. Zusätzlich fallen durchschnittlich 3 Dienste pro Monat an, an denen sowohl der Diensttag als auch der Folgetag nicht für Training zur Verfügung steht. Es sollten 5 Fortbildungstage genutzt werden, und 5 Krankentage pro Jahr werden angenommen. Mit der neuen WBO sollten mindestens 90 Tage für die 200 Endoskopien und zusätzliche Tage für Funktionen wie 400 Sonographien während der Weiterbildung eingeplant werden. Da jeweils 6 Monate für die Rotation in die Ambulanz/Notaufnahme und auf die Intensivstation entfallen, stehen insgesamt 510 Tage für die operative Ausbildung zur Verfügung, was etwa 539 geforderten operativen Eingriffen gegenüber steht. Somit ist der CAJC-Quotient für die alte MWBO von einer WBO-relevanten Operation pro Tag weiterhin knapp gültig, bewegt sich jedoch weiter Richtung 1,5 Eingriffe pro Tag. Eine frühzeitige und geplante operative Ausbildung ist daher entscheidend, um den Anforderungen im gegebenen Zeitrahmen gerecht zu werden.

### Mindestmengen

Die Ergebnisse der Umfrage unterstreichen die absolute und vor allem zeitnahe Notwendigkeit struktureller Änderungen (Rotationen, Kooperationen). Allerdings besteht eine eklatante Lücke zwischen Wunsch und Wirklichkeit, da nur 61 % konkrete Rotationskonzepte (intern/extern) für die operative Weiterbildung angeben. In der aktuellen Diskussion um Mindestmengen [17] und Krankenhausreform [22] liegt der Fokus verstärkt auf der Verteilung komplexer onkologischer Eingriffe (Pankreas, Ösophagus, teilweise kolorektale Eingriffe) sowie der ambulanten Durchführung von Basiseingriffen (proktologische Eingriffe, Herniotomie usw.). Die Stellungnahme der Regierungskommission erwähnt die Weiterbildung von Ärzt:innen lediglich in einem Satz: „Darüber hinaus sollten Level-I-Krankenhäuser auch in Bezug auf die Ausbildung der Ärztinnen und Ärzte fest mit Level-III-Krankenhäusern kooperieren.“ Sie würde eine engere Vernetzung fördern, bei der Teile der Weiterbildung in verschiedenen Einrichtungen durchgeführt werden können, was als positiver Effekt für die Breite der Weiterbildung angesehen wird [22]. Jedoch sind weder spezielle finanzielle Ressourcen für die Weiterbildung geplant, noch gibt es Vorgaben für Kooperationen auf verschiedenen Versorgungsebenen. Dies verdeutlicht, dass die Weiterbildung weiterhin eine untergeordnete (bis gar keine) Rolle spielt und die einzelnen Klinikleitungen – oder im schlimmsten Fall die angestellten WBA selbst – die entsprechenden Kooperationen und Kosten selbst organisieren bzw. tragen müssen. Kurzum: Die Gesundheitspolitik ignoriert die strukturierte Weiterbildung von Ärzt:innen. Dies trifft zum Teil auch auf die weiterbildenden CÄ zu. Die ärztliche Selbstverwaltung muss sich diesbezüglich deutlicher positionieren, um die Qualität der Weiterbildung zu gewährleisten. Dies gelingt vorrangig über eine engere Verzahnung zwischen den Berufsverbänden und den Ärztekammern. Während des Deutschen Chirurgie Kongresses im April 2023 führte die CAJC eine nichtrepräsentative Umfrage zur Auswirkung der aktuellen Krankenhausreform durch. In 62 vollständigen Fragebögen zeigt sich, dass die Teilnehmer:innen aktuell keine Abstimmung der nWBO auf die Krankenhausreform sehen und keine positiven Auswirkungen auf die chirurgische Weiterbildung erwarten. Die Ergebnisse dieser Umfrage deuten auf eine eher pessimistische Stimmung hinsichtlich der chirurgischen Weiterbildung insbesondere im Kontext mit der aktuell geplanten Krankenhausreform hin. Diesbezüglich muss ein Umdenken stattfinden, um dem Mangel an chirurgischem Nachwuchs entgegenzuwirken.

## Optimierung chirurgischer Weiterbildungskonzepte

Die CAJC vertritt die Interessen junger Viszeralchirurg:innen < 40 Jahren, wobei die strukturierte Weiterbildung einen zentralen Schwerpunkt bildet [14, 23]. Dabei werden kontinuierlich Konzepte und Ideen entwickelt, um die chirurgische Weiterbildung zu optimieren, selbst unter wachsendem Kosten- und Zeitdruck [14, 23]. Im Folgenden möchten wir einige dieser Konzepte unter Berücksichtigung der Umfrageergebnisse vorstellen und diskutieren:

### Teilschrittekonzept

**Abb. 6:**
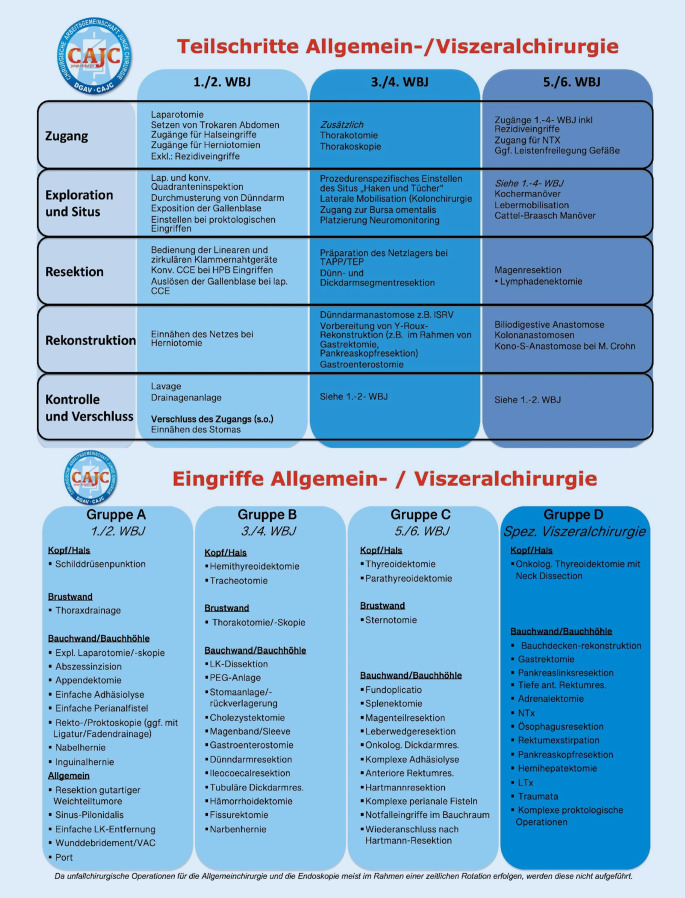
Aktualisierte Teilschrittekarten adaptiert nach Johannink et al. [14]. *WBJ* Weiterbildungsjahr, *LK* Lymphknoten, *CCE* Cholezystektomie, *TAPP* transabdominelle präperitoneale Patch-Plastik, *TEP* totale extraperitoneale Patch-Technik, *ISRV* Ileostomarückverlagerung, *NTx, LTx, PTx* Nieren‑, Leber- und Pankreastransplantation

Um erforderliche Kompetenzen zu erlangen, sollte ein schrittweiser Übergang von Verantwortung und Selbstständigkeit in logischen und aufeinander aufbauenden Stufen, wie im Teilschrittkonzept [13, 14] dargestellt, erfolgen. Eine multizentrische Beobachtungsstudie der CAJC in 21 operativen Zentren zeigte, dass die Anzahl der durch WBA durchgeführten Teilschritte oft überschätzt wurde, was darauf hinweist, dass sie nur begrenzt in den operativen Ablauf eingebunden waren [13]. Eine Studie mit 1747 Schilddrüsenoperationen ergab keinen signifikanten Unterschied in den postoperativen Komplikationen zwischen Eingriffen mit und ohne WBA-Beteiligung [16]. In der Tat wurde in einer Analyse von Raval et al. [21] festgestellt, dass die Beteiligung von WBA mit einer geringeren Sterblichkeitsrate in Verbindung stand. Die Entscheidung darüber, welche Teilschritte assistiert werden können, sollte individuell, abhängig von den Fällen und den Bedürfnissen der WBA getroffen werden. Die für die nWBO aktualisierten Teilschrittekarten ([14]; Abb. 6) können als Richtlinie dienen und dazu anregen, dieses Konzept in den Kliniken zu implementieren. Klar formulierte Kompetenzen und Richtzahlen für die verschiedenen Abschnitte der chirurgischen Weiterbildung sollten unter Berücksichtigung standortspezifischer Bedingungen und Kooperationen festgelegt werden. Laut der vorliegenden Umfrage wird das Teilschrittekonzept deutschlandweit nur in etwa 50 % der Abteilungen umgesetzt, was ein erhebliches Steigerungspotenzial zeigt. Es ist jedoch erfreulich zu sehen, dass das Teilschrittekonzept in die nWBO aufgenommen wurde, indem explizit 50 Laparotomien und deren Verschluss sowie 30 explorative Laparotomien/Laparoskopien gefordert werden, unabhängig von der Operation. Dies verdeutlicht eine Verschiebung der Fokussierung auf vollständige Operationen hin zur Entwicklung chirurgischer Kompetenzen. Angesichts der Verknappung der Zeit im Operationssaal für WBAs ist es von großer Bedeutung, geeignete Strukturen zur Vermittlung fachärztlicher Kompetenzen, z. B. durch das Teilschrittekonzept, zu schaffen.

### Education Team Time Out

Auch andere Konzepte steigern den Lernerfolg: Das intraoperative Gespräch im Operationsteam über Teilschritte und Weiterbildungsstand (Education Team Time Out [ETO]) wirkt sich positiv auf die Weiterbildung aus [19]. Das ETO etabliert eine kurze Überprüfung des Wissensstands und des Operationsverlaufes, in der das Operationsteam die wichtigsten Teilschritte diskutiert [10, 19]. Eine aktuelle Studie zeigte positive Auswirkungen auf das gesamte Team: Insbesondere Anästhesist:innen, WBA, Pflegepersonal und Studierende profitierten; die Einführung des ETO führte zu einem tendenziellen Anstieg assistierter Schritte in der onkologischen Viszeralchirurgie [10].

### Simulationstraining

Konsequentes Simulationstraining könnte das Training praktischer Fähigkeiten optimieren und so die kürzere Weiterbildungszeit im Operationssaal ausgleichen [3]. Leider verfügen nur 35 % der deutschen Kliniken über Simulatoren, die technisch ausgefeilten Geräte befinden sich an UK [6]. Bereits kurzes Üben kann die Bewegungseffizienz erheblich steigern [28]. Die Integration von Simulationstraining in die Weiterbildung wird positiv bewertet; besonders Anreize wie ein „Laparoskopie-Führerschein“ oder Benchmarks [3] fördern die Einführung in die MIC.

Das Üben an robotischen Systemen sollte nicht auf Profis beschränkt sein, um die Lernkurven zu verkürzen [27]. Die Einführung eines strukturierten Kurrikulums für die robotische Chirurgie unter Einbezug von Simulationstraining verkürzt nachweislich die Lernkurve und sollte daher allen angepasst an die Qualifikation in allen Weiterbildungsstufen eingesetzt werden [27]. Dennoch ergab die aktuelle Befragung, dass die robotische Chirurgie nach Meinung der Befragten noch nicht in die WBO aufgenommen werden sollte. Dies bestätigt eine aktuelle Übersichtsarbeit, bei der die robotisch assistierten Eingriffe in der Viszeralchirurgie in Deutschland noch in der deutlichen Minderheit sind [5].

### Mentoring

Der Bedarf an einem strukturierten Weiterbildungskonzept zeigt sich nicht nur bei WBA, sondern bereits während des praktischen Jahres [4]. In dieser Phase gehen viele zukünftige Chirurg:innen verloren, wenn sie zu Beginn ihrer Weiterbildung auf frustrierte WBA treffen, die sich allein gelassen fühlen [4]. Feste Ansprechpartner als Mentor:innen können hier den chirurgischen Nachwuchs fördern und die Integration in die Abteilung stärken. Die strukturierte pädagogische Ausbildung sollte als Kernaufgabe für Chirurg:innen anerkannt werden, wie es zahlreiche „Train-the-Trainer“-Initiativen tun [9], inklusive regelmäßiger Weiterbildungsgespräche, Logbuchführung und strukturierter Operationszuteilung [24].

Die aktuelle Umfrage zeigt, dass insbesondere Frauen die Erreichbarkeit der Richtzahlen pessimistischer einschätzten. Dies wird durch Studien gestützt, die strukturelle Benachteiligungen von Frauen in operativen Fachbereichen aufzeigen [8, 15]. Frauen verlassen die Chirurgie oft aufgrund mangelnder Förderung [18]. Etablierte Mentoringprogramme, wie das der „Die Chirurginnen e. V.“, bieten eine Gelegenheit, potenzielle Fachwechsel zu verhindern und weibliche Rollenvorbilder zu fördern [11].

### Gute Weiterbildung/Zertifizierung

Die Umfrage zeigt, dass die Erreichbarkeit der Richtzahlen signifikant besser bewertet wurde, wenn in der Abteilung ein strukturiertes Weiterbildungskonzept vorhanden war, im Vergleich zu Abteilungen ohne ein solches Konzept. In einer deutschlandweiten Umfrage des Marburger Bundes gaben lediglich 15 % der Ärzt:innen an, dass ihnen ein strukturierter Weiterbildungsplan zur Verfügung gestellt wurde [2]. Aus Sicht der Autor:innen ist die Struktur der Weiterbildung und dabei insbesondere die geplante zielführende Zuteilung zu Weiterbildungseingriffen essenziell. Die Richtzahlen erfordern eine deutlich optimierte Planung der Eingriffe in der Mindestweiterbildungszeit. Nur durch eine kontinuierliche Erfassung der absolvierten Eingriffe und die entsprechende Planung des Einsatzes im Operationssaal ist es möglich, die WBA hier zur Facharztreife zu führen. Dabei ist die große Mehrheit der Umfrageteilnehmer:innen aber auch der Meinung, dass eine gute Weiterbildung ohne Überstunden nicht funktionieren kann, was die Bedeutung auch zusätzlicher Lehr- und Trainingsangebote unterstreicht. Um die Qualität der Weiterbildung zu überprüfen, könnte beispielsweise eine objektive Zertifizierung der Weiterbildung erfolgen, die eine strukturierte Weiterbildung bescheinigt.

## Fazit für die Praxis

- Unabhängig von Versorgungsstufen und Altersgruppen herrscht eine tendenziell pessimistische Stimmung in Bezug auf die Umsetzung der nWBO, insbesondere in Bezug auf das Erreichen der vorgegebenen Ziele innerhalb der üblichen Ausbildungszeit.
- Es bestehen berechtigte Bedenken hinsichtlich der Erreichbarkeit von Richtzahlen im neuen Weiterbildungskatalog und der potenziell verschärfenden Auswirkungen von Mindestmengen und der bevorstehenden Krankenhausreform.
- Die durchgängige Implementierung einer transparenten und strukturierten Weiterbildung ist ein entscheidender Faktor zur Verbesserung der Weiterbildung.
- Rotationen und Kooperationen sind der Schlüssel für die künftige Weiterbildung.
- Bewährte Lehr- und Trainingskonzepte haben weiterhin eine Schlüsselrolle.

## Abkürzungen

CÄ: Chefärzt:innen
CAJC: Chirurgische Arbeitsgemeinschaft Junge Chirurgie
d.F.: Der Fälle
DGAV: Deutsche Gesellschaft für Allgemein- und Viszeralchirurgie
ETO: Education Team Time Out
FÄ: Fachärzt:innen
GV: Grundversorger
k. A.: Keine Angabe
M: Männliche Umfrageteilnehmer
MIC: Minimal-invasive Chirurgie
MV: Maximalversorger
MWBO: Muster-Weiterbildungsordnung
NC: Niedergelassene Chirurgie wie Praxis/MVZ
nWBO: Neue Weiterbildungsordnung
OÄ: Oberärz:innen
RV: Regelversorger
UK: Universitätsklinikum
W: Weibliche Umfrageteilnehmer
WBA: Weiterbildungsassistent:innen
